# Promising Anti-Mitochondrial Agents for Overcoming Acquired Drug Resistance in Multiple Myeloma

**DOI:** 10.3390/cells10020439

**Published:** 2021-02-19

**Authors:** Vanessa Innao, Vincenzo Rizzo, Andrea Gaetano Allegra, Caterina Musolino, Alessandro Allegra

**Affiliations:** 1 Division of Hematology, Department of Human Pathology in Adulthood and Childhood “Gaetano Barresi”, University of Messina, 98125 Messina, Italy; vinnao@unime.it (V.I.); andrea.allegra@hotmail.it (A.G.A.); cmusolino@unime.it (C.M.); 2 Department of Clinical and Experimental Medicine, University of Messina, 98125 Messina, Italy; vincenzo.rizzo@unime.it

**Keywords:** multiple myeloma, drug resistance, mitochondrial-resistance, chemotherapy, apoptosis

## Abstract

Multiple myeloma (MM) remains an incurable tumor due to the high rate of relapse that still occurs. Acquired drug resistance represents the most challenging obstacle to the extension of survival and several studies have been conducted to understand the mechanisms of this phenomenon. Mitochondrial pathways have been extensively investigated, demonstrating that cancer cells become resistant to drugs by reprogramming their metabolic assessment. MM cells acquire resistance to proteasome inhibitors (PIs), activating protection programs, such as a reduction in oxidative stress, down-regulating pro-apoptotic, and up-regulating anti-apoptotic signals. Knowledge of the mechanisms through which tumor cells escape control of the immune system and acquire resistance to drugs has led to the creation of new compounds that can restore the response by leading to cell death. In this scenario, based on all literature data available, our review represents the first collection of anti-mitochondrial compounds able to overcome drug resistance in MM. Caspase-independent mechanisms, mainly based on increased oxidative stress, result from 2-methoxyestradiol, Artesunate, ascorbic acid, Dihydroartemisinin, Evodiamine, b-AP15, VLX1570, Erw-ASNase, and TAK-242. Other agents restore PIs’ efficacy through caspase-dependent tools, such as CDDO-Im, NOXA-inhibitors, FTY720, GCS-100, LBH589, a derivative of ellipticine, AT-101, KD5170, SMAC-mimetics, glutaminase-1 (GLS1)-inhibitors, and thenoyltrifluoroacetone. Each of these substances improved the efficacy rates when employed in combination with the most frequently used antimyeloma drugs.

## 1. Introduction

Multiple myeloma (MM) is a malignant disease caused by plasma cell clonal proliferation in bone marrow, which can cause damage to the kidneys and skeletal fractures, but also involve any organ [1]. The advent of novel drugs has improved the overall survival of myeloma patients. Many experimental studies on innovative treatment strategies, such as vaccines and antagomirs, are proliferating, but the relapse rates remain unacceptably high [2,3]. In real life, combinations of proteasome inhibitors (PIs), immunomodulators, monoclonal antibodies, and anti-B-cell maturation antigen (anti-BCMA) represent the best treatment, but several prognostic factors impact the response rates of these patients. Three-drug combinations, using lenalidomide as the back bone, with carfilzomib (CFZ), ixazomib (Ixa), elotuzumab (Elo), and daratumumab (Dara), are widely used across the world in clinical practice, especially in relapsed/refractory multiple myeloma (RRMM) patients post-first treatment lines with bortezomib (BTZ) [4]. By improving the toxicity profile and effectiveness rates of first-generation compounds, these therapeutic regimes currently represent the best strategies for MM patients, significantly prolonging progression-free survival and overall survival [5]. In addition, the increasing use of antibiotic, antimycotic, and anti-viral prophylaxis, and growth factors, with awareness, and the more serious control of cardiovascular and neurological drugs’ collateral effects have also allowed infective, hematological, cardiological, and ocular complications related to all classes of drugs or some of them to be reduced [6]. Furthermore, infusion/hypersensitivity-related reactions (I/HRRs) to monoclonal antibodies (moAbs) are well-managed by the combination of H1 and H2 antihistamines, with steroids and paracetamol, used as pre-medication in the recommended premeditation schemes.

However, despite improvements in response rates, patients continue to die, and MM is still an unhealthy disease, acquiring aggressivity over the years, with extra-medullar localizations, also in the central nervous system (CNS) or transforming into plasma cellular leukemia. Therefore, in trying to outline the best prognostic drivers of disease since the onset, scientists have identified the minimal residual disease (MRD) negativity as the best marker of a good prognosis [7]. In this scenario, many scientists have focused their studies on intracellular and molecular mechanisms underlying the inadequate response of aberrant plasma cells to the most commonly used drugs. The high proteosynthetic activity of clonal plasma cells was identified as a nodal point in the development of PIs. In fact, the proteasome not only represents an active intracellular complex in protein degradation, but is also responsible for maintaining normal cellular functions. The control system of protein degradation represents an important Achilles heel in many tumor cells. Proteolysis partially begins in cytosol, where the proteins to be degraded are marked by ubiquitins, which are driven within the proteasome. Here, they are cut into small polypeptides or amino acids. Inhibition of the proteasome leads to cellular death, due to the accumulation of ubiquitinated, destructed, or malfunctioning proteins, by determining the development of a toxic intracellular environment, causing antiproliferative and pro-apoptotic effects. In some cell lines, the stabilization of p53, resulting from reduced proteolytic activity, may induce apoptosis. This capability, combined with antiangiogenic and antiproliferative activities, makes PIs potential anticancer agents. However, the prolonged use of PIs leads to adaptation by tumoral cells, because they tend to offset the blockade of the ubiquitin-proteasome system (UPS) using other proteolytic routes.

The UPS has been widely studied, but there are few studies on the stress- or chemical-induced mitochondrial effects, such as cell damage caused by chemotherapy. In this regard, Shadel et al. identified a new function of mitochondria: In response to chemical stress, they activate molecular alarm signals followed by nuclear DNA (nDNA) damage. In previous studies, the researchers showed that cells responded to mitochondrial DNA (mtDNA) changes similarly to an external pathogen, such as a virus, due to the release of mediators capable of activating cell protection programs [8]. Then, they revealed that some anti-tumoral drugs, such as doxorubicin, cause mtDNA and nDNA damage. This causes the expression of Interferon Stimulated Genes (ISG), normally activated by viruses, with the effect of protecting nDNA, often responsible for chemo-resistance. The increased sensitivity of mtDNA, due to the lack of repair tools, makes it a very good DNA stress sensor [9].

Bortezomib (BTZ) and carfilzomib (CFZ) inhibitors of the 26S proteasome in cancer cells, but not in normal cells, represent a powerful back bone in the modern approach to MM patients, although their therapeutic efficacy is often affected by the increasing emergence of an acquired resistance to them. In this regard, with the aim of overcoming these issues, several studies have been conducted. Nelfinavir (NFV), for example, has shown a high capacity to overcome the PI resistance via a voltage-dependent anion channel (VDAC) interaction. These porins represent mitochondrial proteins controlling cross-talk between mitochondria and the rest of the cell, involving cell death, via mitochondria-mediated apoptosis. In this regard, we recently reported the antitumorigenic action of NFV in MM cells resistant to PIs, acute myeloid leukemia (AML), chronic lymphoid leukemia (CLL), and diffuse large B cell lymphoma (DLBCL) [10]. The discovery of new cellular targets and new compounds often saw smaller molecules at the beginning, such as nanobodies and AntagomiRs, which could ensure long-term efficacy and sensitivity [3,11].

**Figure 1:**
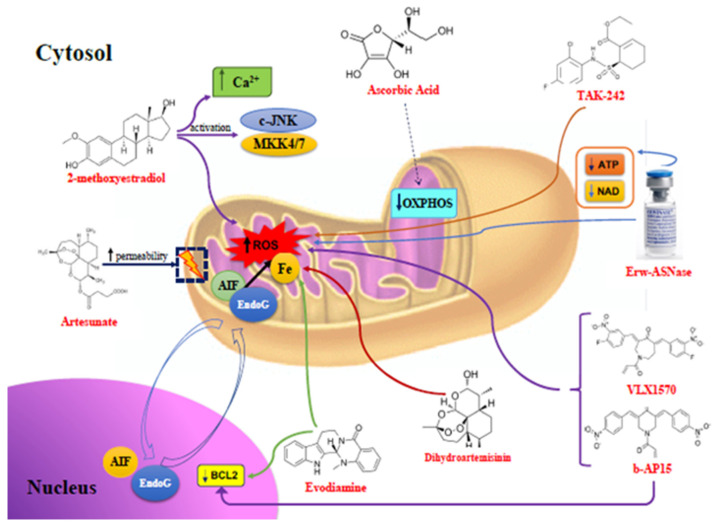
Caspase-independent mechanism of action of anti-mitochondrial agents. Mitochondrial targets of compounds with pro-apoptotic actions on myeloma cells are shown: 2-methoxyestradiol increases mitochondrial ROS production and increases Ca2+ levels in the MM cells, also causing JNK and MKK4/7 activation; artesunate increases the mitochondrial membrane permeability and ROS concentration in MM cells, inducing mitochondrial protein transfer into the nucleus, which causes DNA damage, AIF, and EndoG activation; ascorbic acid reduces cell respiration by the direct inhibition of mitochondrial oxidative phosphorylation in MM cells; DHA induces a mitochondrial Fe2+-dependent ROS increase, also inhibiting BCL2 when associated with Dexa; Evo activates mitochondrial pathways and inhibits nuclear BCL2 with an ROS increase; b-AP15 overcomes BTZ-resistance, inducing oxidative stress via ROS, through the induction of chaperones HSP70B’ and HSP40, then resulting in Nrf-2 and its target HO-1 induction; VLX1570 determines cell oxidative stress, inducing the accumulation of polyubiquitin chains, causing MM cell death in combination with Dexa in vivo; Erw-ASNase in combination with CFZ causes MM cell death, increasing ROS generation and Nrf2 deregulation, and reducing the ATP and NAD intracellular content; TAK-242 associated with BTZ generates an ROS and RNS increase, causing MM cell death. *Abbreviations*: ROS, Reactive Oxygen Species; JNK, c-Jun N-terminal Kinase; MKK4/7, Mitogen-activated protein Kinase Kinase 4/7; MM, multiple myeloma; AIF, Apoptosis Inducing Factor; EndoG, Endonuclease G; OXPHOS, Oxidative Phosphorylation; DHA, Dihydroartemisinin; BCL2, B-Cell Lymphoma 2; Dexa, Dexamethasone; Evo, Evodiamine; b-AP15, deubiquitinase inhibitor b-AP15; BTZ, Bortezomib; HSP70B’, Heat Shock Protein 70B’; HSP40, Heat Shock Protein 40; Nrf-2, Nuclear factor erythroid 2-Related Factor 2; HO-1, Heme Oxygenase 1; VLX570, deubiquitinase inhibitor VLX570; Erw-ASNase, L-Asparaginase purified from *Erwinia chrysanthemi*; CFZ, carfilzomib; ATP, Adenosine TriPhosphate; NAD, Nicotinamide adenine dinucleotide; TAK-242, Toll-like receptor 4 Antagonist 242 or Resatorvid; and RNS, reactive nitrogen species.

**Figure 2:**
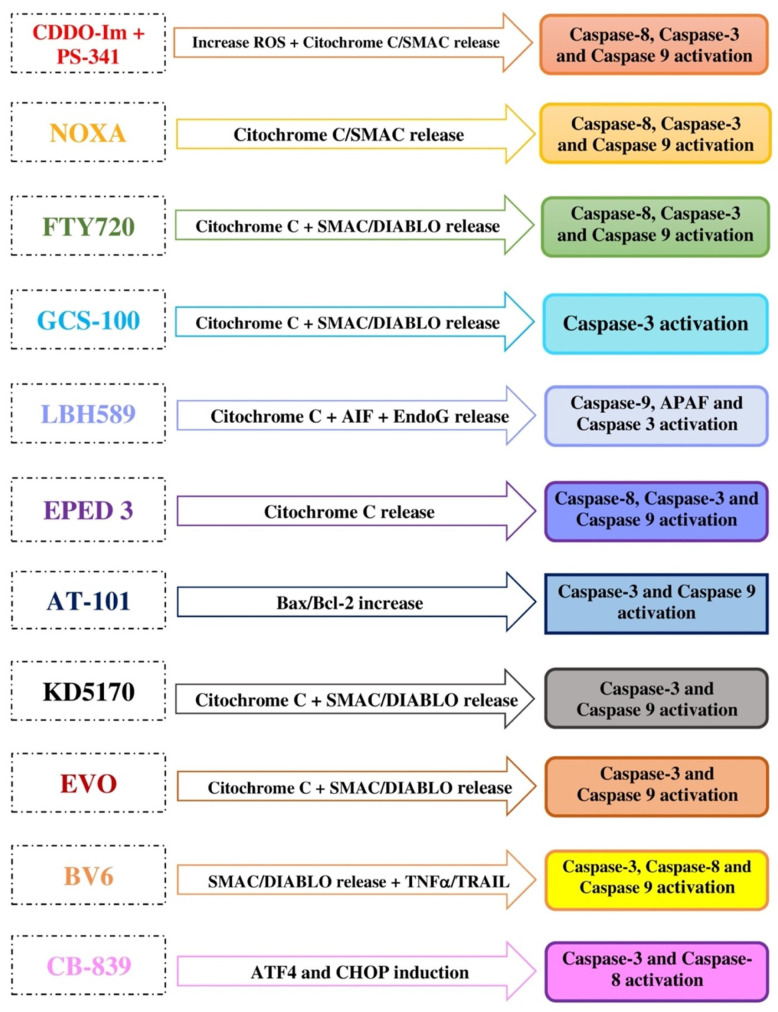
Anti-mitochondrial agents causing caspase-dependent apoptosis. CDDO-Im associated with PS-341 induces apoptosis in multidrug-resistant MM cell lines targeting several pathways, such as caspase-8, capsase-3, and caspase-9 activation and mitochondrial cytochrome c and SMAC release into the cytosol, but also via ROS; NOXA causes MM cell death, inducing cytochrome c release into the cytosol, followed by a proteolytic cascade involving caspase-9, -3, and -8; FTY720 with Dexa and anti-Fas antibodies reverts MM cells’ drug resistance, causing an increase in the permeability of the mitochondrial membrane, followed by cytochrome c and SMAC/DIABLO release into the cytosol, and activates proapoptotic Bax, resulting in the activation of caspase-8, -9, and -3; GCS-100 induces caspase-3 activation in BTZ-resistant MM cells via cytochrome c and SMAC/DIABLO release into the cytosol; LBH589 causes apoptosis in MM drug-resistant cells via caspase-independent and -dependent mechanisms, principally due to an increase in the mitochondrial membrane permeability, release of cytochrome c into the cytosol, activation of apoptosome (made up of caspase-9, APAF-1, and caspase-3), and then the release of AIF and EndoG; EPED3 determines MM drug-resistant cell death, destroying the mitochondrial membrane, followed by cytochrome c release into the cytosol, with the final activation of caspase-8, caspase-9, and caspase-3; AT-101 increases the Bax/BCL-2 ratio, inducing apoptosis via caspase-3 and caspase-9 activation; KD5170 enhances MM cell death of BTZ, causing loss of the mitochondrial membrane integrity, followed by cytochrome c and SMAC/DIABLO release into the cytosol, with caspase activation; similarly, Evo in combination with BTZ blocks MM cell proliferation, due to caspase-3 and -9 activation; BV6 induces the degradation of IAPs, sensitizing MM cells to death ligands TNF-a and TRAIL-induced cell death, activating the caspase pathway; CB-839, in combination with BTZ, Ixa, Opro, or even better with CFZ, causes MM cell death due to induction of the ER stress markers ATF4 and CHOP, and caspase-3 and -8 activation. Abbreviations: CDDO-Im, triterpenoid 2-cyano-3, 12-dioxooleana-1, 9-dien-28-oic acid, Imidazoline; PS-341, Bortezomib; MM, multiple myeloma; SMAC, Second mitochondria-derived activator of caspases; ROS, Reactive oxygen species; NOXA, phorbol-12-myristate-13-acetate-induced protein 1; FTY720, Fingolimod; Dexa, Dexamethasone; Fas, Fas cell surface death receptor; SMAC/DIABLO, Second mitochondria-derived activator of caspases/direct inhibitor of apoptosis-binding protein with low isoelectric point; Bax, Bcl-2 Associated X; GCS-100, apoptosis inducer GCS-100; BTZ, Bortezomib; LBH589, Panobinostat; APAF-1, Apoptotic protease activating factor-1; AIF, Apoptosis inducing factor; EndoG, Endonuclease G; EPED3, 9-dimethyl amino-ethoxy ellipticine; AT-101, Gossypol; Bax/BCL-2, BCL-2-associated X protein/B-Cell Lymphoma 2; KD5170, mercaptoketone-based histone deacetylase inhibitor; Evo, Evodiamine; BV6, SMAC mimetic BV6; IAPs, Inhibitors of Apoptosis; TNF-a, Tumor Necrosis Factor-alpha; TRAIL, TNF-Related apoptosis-inducing ligand; CB-839, glutaminase 1, GSL1, inhibitor or Telaglenastat; Ixa, Ixazomib; Opro, oprozomib; CFZ, carfilzomib; ATF4, Activating Transcription Factor 4; and CHOP, C/EPB Homologous Protein.

Focusing on proteotoxic stress and oxidative damage in the tumoral cells, whose survival is based on the activation of antiapoptotic mechanisms, or the inhibition of pro-apoptotic tools, in this review, we want to describe the most interesting mechanisms for the re-sensitization of myeloma cells to the action of the most commonly used drugs, through the induction of mitochondrial stress and caspase-independent and -dependent apoptotic mechanisms (Figure 1 and Figure 2). During the last few decades, several studies on the centrality of a mitochondrion in the acquired drug resistance in multiple myeloma have emerged [12,13].

A recent report described massive metabolic changes in BTZ and carfilzomib-resistant MM cells, including the activation of antioxidant mechanisms, such as the regeneration of glutathione and nicotinamide adenine dinucleotide phosphate (NAD(P)H), a high oxidative phosphorylation and tricarboxylic acid cycle, a shift to lipogenesis and amino acid biosynthesis, and ab increase in sphingomyelin synthesis and protein folding [14]. Finally, the authors showed that Venetoclax, which is a B-cell lymphoma 2 (BCL-2) inhibitor, and compound C, which is an AMP-activated protein kinase (AMPK) inhibitor, were more effective in BTZ- and CFZ-resistant cells, due to their potent effects on mitochondria. Using tricyclodecan-9-yl-xanthogenate (D609), which is an inhibitor of sphingomyelin synthase with known antiviral and antitumor properties, they proved the importance of sphingomyelin synthesis in PI-resistance, demonstrating a consistent synergic effect of D609 in combination with BTZ and CFZ in MM cells [14].

Considering these pathways, based on our knowledge, our report represents the first collection of several compounds targeting the mitochondria that are able to overcome drug resistance in MM.

## 2. Re-Sensitization to Chemotherapy through Caspase-Independent Apoptosis via ROS

Despite therapeutic advances, and sometimes regardless of the prognostic risk, multiple myeloma patients still experience relapses after exposure to several agents and reduced survival [15]. The induction of cellular oxidative damage has opened the way to the study of new compounds that kill plasma cells with acceptable safety profiles, and we can consider some of them as promising adjuvants in the treatment of MM in clinical practice. Some of these molecules act as anticancer drugs by direct mitochondrial damage, while others determine wider cellular changes followed by mitochondrial changes.

The mitochondrial function plays a central role in the cellular reactive oxygen species (ROS) balance and, in this regard, to overcome bortezomib-resistance in MM cell lines, in 2013, Korean researchers identified a compound active via ROS overproduction [16]. Compared with BTZ alone, representing the first PI available for MM patients, they showed that 2-methoxyestradiol (2ME), which is a natural estrogen metabolite, induced a higher rate of MM cell death, due to increases in the mitochondrial ROS and Ca^2+^ levels in the cells, the activation of c-Jun N-terminal kinase (JNK), and mitogen-activated protein kinase kinase 4/7 (MKK4/7) [17]. The capability of 2ME to increase the death of cancer cells has also been proved to be powerful against other types of tumor, such as ovarian, lung, breast, and colorectal tumors [13,18,19,20,21].

In 2014, scientists from Little Rock explained the mechanism inducing apoptosis by Artesunate, which is a well-tolerated anti-malarial drug. Targeting mitochondria and increasing their membrane permeability in MM cell lines, due to an increased bivalent iron-dependent mitochondrial production of ROS levels, this compound turned out to be an effective cellular death stimulator, through non-caspase-mediated apoptosis, such as in lung adenocarcinoma cells [21,22]. The osmotic swelling of the mitochondrial matrix and compression of vesicles caused release into the cytosol and subsequent migration into the nucleus of pro-apoptotic proteins, such as cytochrome c, apoptosis inducing factor (AIF), and endonuclease G (EndoG). The role of EndoG in cell death is still unclear, whereas AIF, binding directly to DNA, causes chromatin condensation and DNA fragmentation. In addition, Artesunate has been shown to cause cell death independently of bortezomib-resistance, increasing its effectiveness [23].

The importance of cytoplasmic iron assessment in MM cells was underlined by Tricot el al. in 2017. Comparing the gene expression profiles (GEPs) of 18 mitochondrial genes in 22 normal plasma cells from 44 monoclonal gammopathy of undetermined significance (MGUS) patients and 351 newly diagnosed MM (NDMM) patients during a total therapy trial, they demonstrated that higher cytosolic iron levels promote cell proliferation and induce acquired drug resistance, due to its influence on the expression of many mitochondrial genes, including mitochondrial biogenesis signatures and oxidative phosphorylation. Due to inhibiting mitochondria oxidative phosphorylation and inducing MM cell death, ascorbic acid could become an old but revolutionary compound in synergic treatment in bortezomib-refractory MM patients [24].

Iron and ROS are also involved in the effectiveness of inducing the mitochondrial apoptotic pathway of Dihydroartemisinin (DHA), which is a derivative of artermisin (ART), employed for malaria treatment. Interacting with ferrous ions (Fe^2+^) and oxygen to produce ROS, DHA has been proven to be active in promoting myeloma cell death, overcoming the Dexamethasone resistance by reverting its action to upregulate the expression of the BCL2 protein. In addition, the high efficacy of DHA+Dexa in vitro and in vivo is due to mitochondrial cytochrome C translocation to the cytoplasm, resulting in caspase-mediated apoptosis, leading to high hopes regarding the therapeutic success of this substance [25]. In addition, DHA has exhibited a high anti-tumoral efficacy on MM cells by favoring autophagy and inhibiting angiogenesis, through reducing vascular endothelial growth factor (VEGF) levels [26,27].

An herbal substance from traditional Chinese medicine called Evodiamine (Evo) has displayed great anticancer activity in several tumor types [28,29,30,31]. Derived from the drying mature fruit of *Evodia rutaecarpa*, Evo induces apoptosis in MM cells, activating several pathways, including BCL2 inhibition and ROS accumulation. Similar to other compounds, Evo treatment increases mitochondrial cytochrome c release into the cytosol, upregulating ROS levels, due to mitochondrial superoxide production, and activating intrinsic mitochondrial apoptosis pathways [32]. Evo’s toxicity profile has still not been well-defined by giving cardiac arrhythmias and prolonging the bleeding times, although studies testing the acute toxicity seem to provide promising data on Evo’s development as a new anticancer remedy [33].

Additionally, deubiquitinase inhibitors (DUBs), such as *b-AP15,* have been shown to overcome the acquired resistance to BTZ in MM, Waldestrom macroglobulinemia (WM), and diffuse large B cell lymphoma (DLBCL), resulting in mitochondrial dysfunction [34,35,36]. Inducing oxidative stress in MM cells, b-AP15 revealed high antiproliferative activity, causing mitochondrial deformations, through the induction of the chaperones heat shock protein 70B’ (HSP70B’) and heat shock protein 40 (HSP40), resulting in nuclear factor erythroid 2-related factor 2 (Nrf-2) and its target heme-oxygenase 1 (HO-1) induction, but without lipid peroxidation [37]. The caspase-independent pro-apoptotic effects of b-AP15 were very high in tumor cells overexpressing BCL2 family proteins and defective in Tumor Protein p53 (TP53) [38], not just in MM [39,40,41,42,43,44]. However, these data have only been produced from cell line studies, and should be confirmed by in vivo tests.

Another recognized competitive DUB capable of inducing apoptosis in MM cells is *VLX1570*, which is a more potent and soluble analogue of b-AP15 able to strongly inhibit Ubiquitin carboxyl-terminal hydrolase 14 (UPS14) activity, inducing the accumulation of polyubiquitin chains, the expression of the chaperone HSP70B’, the oxidative stress marker heme oxygenase 1 (Hmox-1), and apoptosis in MM cells. A GEP study revealed that VLX1570 determines cell oxidative stress similar to b-AP15, in respect to which it has been shown to be more effective because of the best administration [45]. In vivo, a phase I/II clinical trial (NCT02372240) evaluated the efficacy and safety of this compound and Dexamethasone in relapsed/refractory MM (RRMM) patients. Although the efficacy was promising, its low safety profile, which resulted in death by respiratory failure of two of the 14 enrolled patients, caused the premature closure of the study, focusing on the identification of better tolerated substances [46].

Italian researchers recently reported mitochondrial damage and increased ROS generation by L-Asparaginase purified from *Erwinia chrysanthemi* (Erw-ASNase), which is a powerful enhancer of the carfilzomib response in resistant MM cells. Recognizing amino acid depletion as an instrument to better hit tumor cells, the authors analyzed the concept of amino acid starvation, induced by Erw-ASNase. In combination with Carfilzomib, Erw-ASNase caused cell death via increased mitochondrial oxidative stress, due to higher ROS generation, Nrf2 upregulation, and a reduced adenosine triphosphate (ATP) and nicotinamide adenine dinucleotide (NAD) intracellular content [47]. Since *ERW-ASNase* has been approved and is commonly used in clinical practice in the treatment of pediatric acute lymphoblastic leukemia, where the safety profile is fairly high, its tolerability in adult patients is still low, so in vivo studies of multiple myeloma patients are required to assess its applicability as an antimyeloma drug.

A recent report of Italian researchers sustained mitochondrial involvement in BTZ resistance in MM cells, due to the increased signal of Toll-like receptor 4 (TLR4). Combining BTZ with *TAK-242* (Resatorvid), which is a selective TLR4 inhibitor, they overcame MM cell resistance, generating higher oxidative stress due to an ROS and reactive nitrogen species (RNS) increase, followed by depolarization of the mitochondrial membrane and cytochrome c release into the cytosol, finally resulting in the activation of caspase-9 [48].

Often, several cellular death mechanisms are compenetrated, without allowing clear distinctions. Frequently, in fact, the same substances may activate multiple mechanisms capable of killing tumor cells at the same time.

## 3. Re-Sensitization to Chemotherapy through Caspase-Dependent Apoptosis

Acquired resistance to proteasome inhibitors is not only based on the development of caspase-independent anti-apoptotic mechanisms; they also lose their effectiveness when tumor cells gain resistance to caspase-dependent mechanisms.

In this regard, an old report published in 2004 identified the role of low-dose *CDDO-Im* (triterpenoid 2-cyano-3, 12-dioxooleana-1, 9-dien-28-oic acid, Imidazoline) associated with *PS-341*, which is a bortezomib-proteasome inhibitor, in the induction of apoptosis in MM cells resistant to conventional therapy, including doxorubicin, melphalan, and dexamethasone. Studies on leukemia and lymphoma cells have demonstrated that CDDO-Im induces apoptosis in several ways [49,50,51], such as through the activation of caspase-8 and -3, and the release of mitochondrial cytochrome c. This study showed that PS-341, in combination with low-dose CDDO-Im, exhibited high anti-MM activity in BTZ-resistant MM cell lines and MM patients, without effects on normal lymphocytes, even due to mitochondria-dependent apoptotic signaling pathways. Their effectiveness in cell death included the rapid depletion of mitochondrial glutathione, generation of ROS, loss of mitochondrial membrane potential, release of mitochondrial cytochrome c and the second mitochondria-derived activator of caspases (SMAC) into the cytosol, and activation of caspase-9 and caspase-3. Moreover, mitochondria-independent apoptosis was also described, via the death receptor-mediated-caspase-8-caspase-3 pathway [52]. However, in vivo data refer to studies in murine models, so phase 1 studies are required to establish its applicability in the anticancer therapeutic strategy.

In 2005, the same targeted pathways were described by protein phorbol-12-myristate-13-acetate-induced protein 1 (*NOXA*), which is responsible for the mitochondrial-based apoptotic resistance to PIs, based on the release of cytochrome c, followed by a proteolytic cascade involving caspase-9, -3, and -8. The scientists were able to overcome the PI resistance via NOXA induction, which was p53-independent, because its block, using an antisense oligonucleotide, caused a 30% to 50% reduced apoptotic response in melanoma and myeloma cells, suggesting a therapeutic role of agents triggering NOXA induction in these patients [53]. This pathway is involved in the sensitivity to apoptosis in several types of cancer, such as mantle cell lymphoma and small cell lung cancer [54,55].

In 2005, Swiss researchers reported results on an immunomodulator agent, named Fingolimod (FTY720), which has been approved by the FDA as an oral compound to treat relapsed multiple sclerosis. This agent demonstrated high antitumoral activity in micromolar concentrations in drug-sensitive and -resistant MM cell lines and MM patients, due to the well-known activation of proapoptotic Bax and depolarization with an increased permeability of the mitochondrial membrane, followed by cytochrome c and SMAC/direct inhibitor of apoptosis-binding protein with low isoelectric point (DIABLO) translocation into the cytosol, finally resulting in the activation of caspase-8, -9, and -3. In combination with dexamethasone and anti-Fas antibodies, FYT720 improved the response rates, managing to overcome the acquired drug resistance [56]. Recently, Chinese scientists confirmed these results, proving the synergic action of Fingolimod with metformin that is able to induce apoptosis in MM cells [57]. Despite the study being conducted on MM cell lines, the safety profiles of both compounds are well-known, so there would appear to be no limits in testing them in vivo in MM patients.

The activation of the same mitochondrial targets was described in a report of 2005 on *GCS-100*, which is a derivative citrus pectin polysaccharide, developed for the treatment of several cancers. Alone or in combination with dexamethasone, this compound induced apoptosis in BTZ-resistant MM cells, activating mitochondrial apoptotic signals via the release of cytochrome c and SMAC/DIABLO, followed by caspase-3 induction [58]. However, data in the literature stops 10 years ago and this compound has not been developed, so it cannot be included among the potential drugs that may be expected to treat MM patients in the near future.

For in vitro and in vivo MM cells, caspase-dependent cell death has been observed for LBH589 Panobinostat (LBH589), a hydroxamic acid-derived Hystone Deacetylase (HDAC) inhibitor, which has been proven to display a potent antiproliferative action, showing promising results in myeloid leukemia [59]. In MM, LBH589 induces apoptosis via caspase-independent and -dependent mechanisms, increasing the action of the most frequently used antimyeloma drugs, including bortezomib, melphalan, and dexamethasone. Apoptosis occurs because LBH589 increases the mitochondrial outer membrane permeability, resulting in the release of cytochrome c into the cytosol, which is then responsible for the activation of the apoptosome, made up of caspase-9, APAF-1, and caspase-3, and the release of AIF and EndoG. These results supported the clinical use of LBH589 as a good partner for other antimyeloma drugs, so the FDA approved it in 2014 for the treatment of patients with RRMM after at least two previous therapy lines [60]. However, its low safety profile led clinics not to prefer this choice, so it is an orphan drug in Europe.

A few years later, in 2008, a study on the pro-apoptotic effects of a hydrophilic derivative of the plant alkaloid of Ellipticine (EPED3, 9-dimethyl amino-ethoxy ellipticine) in MM cell lines came to light. Although it is not yet clear whether the mitochondrion is the only or main cell target of EPED3 in the re-sensitization of myeloma cells to chemotherapy, its effectiveness is clear. In fact, intercalating between DNA base pairs and inducing G_2_-M-phase cycle arrest, EPED3 proved to be a powerful anti-myeloma agent in cells with CDC28 protein kinase regulatory subunit 1B (CKS1B) overexpression, which is a cell cycle regulator, even by the inhibition of topoisomerase II and P-glycoprotein-mediated multidrug resistance, without damage to stromal cells [61]. Its effectiveness also appeared to be high in BTZ- and dexamethasone-resistant cell lines, by destroying the mitochondrial membrane, followed by cytochrome c release into the cytosol, with the final activation of caspase-8, caspase-9, and caspase-3. Furthermore, using electron microscopy, the authors showed further ultrastructure changes due to apoptosis activation by EPED3 on MM cells, such as the destruction of mitochondrial inner cristae, nuclear pyknosis, and formation of multiple cytosolic vacuoles, hoping for its use in vivo as an antimyeloma agent in combination with conventional drugs [62]. However, in vitro studies are still ongoing regarding the identification of other effective water-soluble derivatives of Ellipticine, such as anticancer agents and immunomodulators for its low bioavailability [63].

In the same year, Rochester researchers provided similar results on AT-101, which is an R-(−)-gossypol small molecule active in MM resistant-cells in vivo and in vitro, mimicking the BH3 domain of cellular BCL-2 inhibitors. They proved that AT-101 decreased cell proliferation, increasing the Bax/BCL-2 ratio and activating apoptosis via mitochondrial pathways. Caspase-3 and caspase-9 activation resulted from mitochondrial membrane depolarization. Finally, AT-101 in combination with dexamethasone exhibited synergistic toxicity on MM cell lines extracted from MM patients [64]. In combination with Rituximab or Lenalidomide, AT-101 has shown a good efficacy in patients with chronic lymphocytic leukemia (CLL), but no study has yet been started in MM patients and its tolerability profile has not been well-defined (NCT00286780) [65]. In this regard, by enhancing the Noxa-mediated activation of Bak, followed by an increase in the mitochondrial apoptotic pathway, the BH3-mimetic GX15-070 demonstrated synergic activity with bortezomib in mantle cell lymphoma, while ABT-737 induced apoptosis in small cell lung cancer via Noxa/Mcl1 [54,55]. The ABT-analogous 737, 263, and 199 are other Bcl-2-specific BH3-mimetics active against the subgroup of t(11;14) MM patients presenting a Bcl2high/Mcl-1 low profile [66,67,68].

Caspase-3, caspase-8, and caspase-9 are also activated by *KD5170*, which is a mercaptoketone-based histone deacetylase inhibitor that has shown a high antiproliferative efficacy in MM cell lines. An article of 2008 reported the proapoptotic effect of this compound, caused by loss the mitochondrial membrane integrity, with cytochrome c and SMAC/DIABLO release into the cytosol, and thus apoptosis inducing factor activation. In association with BTZ, KD5170 enhanced its antimyeloma effectiveness [69]. In addition, KD5170 exhibited a broad spectrum of antitumoral activity in vitro and in vivo [70], and similar results emerged about another histone deacetylase inhibitor, named *PXD101*, capable of improving the antimyeloma effect of BTZ by the induction of oxidative stress and DNA damage [71]. Both compounds have not yet been tested in patients, so their real applicability as antimyeloma pharmacological agents cannot be established.

Similarly, in 2013, Chinese scientists supported the efficacy of gossypol in inducing apoptosis in myeloma cells in vitro and in vivo, by the inhibition of BCL-2/BCL-XL and caspase-3 and -9 activation [72]. Used as a male contraceptive, the safety data already obtained from its marketing would reduce the test times required to prove its effectiveness in MM patients.

Another natural derivative, called Evodiamine (Evo), is known for its anticancer activity in numerous tumor cells [28,29,30,31], including myeloma. Already mentioned as being responsible for cell damage by ROS, in MM, Evo also selectively blocks cell proliferation and increases apoptosis, vigorously activating caspase-3 and -9. In combination with BTZ, in a xenografted mice model, Evo enhances MM cell death, with a strong reduction in the cell viability, with respect to a BTZ single agent [32], but no data on MM patients are available and there are no negligible data on adverse events related to its use, such as cardiac arrhythmias and prolonged bleeding times, so further in vivo studies are necessary.

Caspase-dependent apoptosis is involved, along with independent mechanisms, in the abovementioned *DHA*, which is highly effective in association with dexamethasone in MM cells, due to mitochondrial cytochrome C translocation to the cytoplasm [24] and this action is valid in many types of cancer [73,74,75,76].

SMAC-mimetics are novel anticancer compounds developed to simulate the endogenous SMAC/DIABLO, which is an inhibitor of a class of proteins known as inhibitors of apoptosis proteins (IAPs). In this way, SMAC/DIABLO acts as an enhancer of the activation of caspases [77,78]. Drug resistance in MM is largely attributed to a reduction in SMAC release [79]. In recent years, fervent attention has been paid to these molecules because of their promising efficacy in several types of tumor [80,81,82,83,84]. In MM cells the novel bivalent SMAC mimetic *BV6* induces the degradation of IAPs (cIAP1 and cIAP2), resulting in the apoptotic death of resistant cell lines via non-canonical NF-kB pathway activation. In the presence of cytochrome c, BV6 sensitizes MM cells to death ligands tumor necrosis factor-a (TNF-a) and TNF-related apoptosis-inducing ligands (TRAIL)-induced cell death, activating the caspase pathway. The sensitizing effect of BV6 on recombinant-TNF-a and killer TRAIL allows us to consider it a modern therapeutic tool in combination with conventional drugs in different MM cells [85], as well as in acute myeloid leukemia [86,87], chronic lymphocytic leukemia [88], and some solid tumors [89,90,91].

Using SMAC-mimetics, in recent years, several reports have underlined the role of controlling apoptosis by IAPs in multiple myeloma. In this regard, the administration of LCL161 has significantly reduced X-linked inhibitor of apoptosis protein (XIAP) activity and cellular inhibitor of apoptosis protein-1 (cIAP1) levels in both sensitive and resistant myeloma cells. In addition, LCL161 determines the up-regulation of the Janus kinase 2/Signaling transducer and activator of transcription (Jak2/Stat3) signaling pathway in resistant cells, showing synergic antimyeloma activity when used with specific Jak2 inhibitors, both in cell lines and patient cells. Finally, associated with death-inducing ligands, LCL161 re-sensitized MM cells to both Fas cell surface death receptor (FAS-L) and TNF-related apoptosis-inducing ligand (TRAIL) [92]. However, these are in vitro studies or preclinical models, and in vivo tests are required to understand the therapeutic impact of these compounds in the treatment of MM patients in the future.

Another SMAC-mimetic molecule with similar inducing apoptosis effects in drug-resistant MM cells is *LBW242*, which is a well-tolerated compound that acts via caspase-8, -9, and -3 activation. When combined with BTZ or melphalan, LBW242 improved the response rates synergistically [76].

The evolution of PIs with second-, third-, and fourth-generation Carfilzomib (CFZ), Ixazomib (Ixa), and Oprozomib (Opro) has improved response rates, but has not resolved the problem of acquired resistance in MM cells. A 2016 study showed that the mechanisms of MM cells based on a loss of susceptibility to CFZ are similar to those observed with BTZ. Specifically, using glutamine as the main energy source, resistant cells appeared to exhibit higher mitochondrial respiration. This assumption has allowed us to use glutaminase-1 (GLS1) inhibitors to overcome the PI resistance of MM cells. Therefore, *CB-839 (Telaglenastat*) in combination with BTZ, Ixa, Opro, or even better with CFZ, has been shown to be a powerful inducer of apoptosis, by intense induction of the ER stress markers Activating Transcription Factor 4 (ATF4) and C/EPB Homologous Protein (CHOP), and caspase-3 and caspase-8 activation [93,94]. Furthermore, GLS selective inhibitors have also displayed good anti-cancer activity that is able to proliferate studies in other tumor cells, as well as B cell lymphoma, breast, liver, ovarian, pancreatic, colorectal, liver, and non-small cell lung cancers [95,96,97,98,99].

According to what has been said so far, the results obtained from a very recent study on the action mechanisms of Venetoclax, which is a BCL-2 antagonist highly effective in t(11;14) MM cells, are not surprising. Its effectiveness is based on several cellular targets, mostly involving the mitochondrion, which showed a reduced respiration in sensitive cells. Using inhibitors of electron transport chain (ETC) Complex I and II, *IACS-010759,* and thenoyltrifluoroacetone (*TTFA*), respectively, the authors demonstrated that the same ETC activity could represent a predictor and a target for Venetoclax sensitivity in MM patients [68].

The mechanisms of action and pathways activated by the various compounds considered in our manuscript are described in Table 1.

## 4. Conclusions

This comprehensive collection of data present in the literature is born from the desire to refine our weapons to improve the response rates and overall survival of multiple myeloma patients, who too often experience relapses [100]. Improvement of diagnostic definitions and the adoption of the minimum residual disease as the best prognostic marker available are not sufficient for permanently healing this disease [7,101]. Therefore, many studies are being carried out to find compounds and mechanisms for the circumvention of this tumor intelligence. Indeed, over the last few years, MM has represented one of the cancers that has progressed the most in terms of survival, mainly thanks to the advent of modern monoclonal antibodies (moAbs), such as Daratumumab and Isatuximab, and anti-BCMA, such as Belantamab mafodotin; however, they often do not exceed cytogenetic high risk [102].

We have reported several substances, also of natural origin, such as Chinese herbs or those from common plants, effective in improving or restoring the response to the most commonly used drugs, as well as PIs, such as BTZ, CFZ, and ixazomib, but no data are available for the abovementioned novel anti-myeloma agents, whose resistance mechanisms remain largely unknown.

In this respect, some of the abovementioned molecules appear, more than others, to be promising adjuvant agents for the therapy of MM patients, such as 2ME, Artesunate, Ascorbic Acid, Fingolimod, Panobinostat, AT-101, Telaglenastat, and Venetoclax. For the others, which are Evodiamine, bAP15, VLX1570, ERW-ASNase, TAK-242, CDDO-Im, GCS-100, EPED3, KD5170, PXD101, BV6, and LBW242, additional preclinical and in vivo tests are required to prove their applicability in clinical practice.

Therefore, we believe that it is necessary to extend research on these compounds, perhaps by testing the same substances that have proved to be effective until now or by finding even better substances.

## Figures and Tables

**Table 1:** Mechanisms of action and pathways activated by anti-mitochondrial agents.

Compounds	Mitochondrial Changes	Pathways Activated	Tumors
2ME	↑ Mitochondrial ROS and Ca^2+^	c-JNK and MKK4/7	MM, ovarian, lung, breast, and colorectal cancers
Artesunate	↑ Mitochondrial ROS, loss of mitochondrial membrane integrity, release of cytochrome c, AIF, and EndoG into the cytosol	Chromatine condensation and DNA fragmentation by AIF and EndoG	MM, AML, melanoma, osteosarcoma, pancreas, breast, prostate, ovarian, renal, CNS cancers
Ascorbic Acid	↓ Iron levels, inhibition of mitochondrial OXPHOS, ↓ ATP	↓ Nrf-2, p53 upregulation, cell cycle arrest	MM, lung, pancreas, breast, cervix, urothelial cancers, and mesothelioma
DHA	↑ Mitochondrial ROS, ↓ Iron levels, ↓ VEGF, loss of mitochondrial membrane integrity, release of cytochrome c into the cytosol	↓ Bcl-2, ↑ caspases activity	MM, neuroblastoma, cervix, liver, pancreas, prostate cancers
Evo	↑ Mitochondrial ROS, loss of mitochondrial membrane integrity, release of cytochrome c into the cytosol	↓ Bcl-2, ↑ caspases 3 and 9 activity, activation of Cdc2/Cyclin B, ↑ IAPs, ↓ NF-kB, ↓ Cyclin D1	MM, T cell leukemia, melanoma, Cervix, colorectal, lung, breast, prostate cancers
b-AP15	↑ Mitochondrial ROS, mitochondrial deformations, ↑ HSP70B’ and HSP40	↓ Bcl-2, ↓ Nrf-2 and HO-1	MM, WM, DLBCL, AML, pancreas, lung cancers
VLX1570	↓ UPS14, ↑ HSP70B’	↓ HO-1, ↓ NF-kB, ↑ caspases activity	MM, WM, ALL, MCL, Ewings Sarcoma, ovarian cancer
Erw-ASNase	↑ Mitochondrial ROS, ↓ mitochondrial ATP and NAD levels, ↓ amino acids	↓ Nrf-2, ↓ genomic instability, ↓ DNA-repair tools	MM, ALL, AML, CML, NK/T cell lymphoma, colon and CNS cancers
TAK-242	↑ Mitochondrial ROS and RNS, mitochondrial membrane depolarization, release of cytochrome c into the cytosol	↓ TLR4, caspase 9 activation	MM, breast, ovarian cancers
CDDO-Im	↑ Mitochondrial ROS, ↓ mitochondrial glutathione, mitochondrial membrane depolarization, release of cytochrome c and SMAC/DIABLO into the cytosol	Induction of caspases 8, 3, and 9	MM, leukemia, lymphoma
FTY720	↑ Mitochondrial ROS, mitochondrial membrane depolarization, release of cytochrome c and SMAC/DIABLO into the cytosol	↑ proapoptotic Bax, activation of caspases 8, 9, and 3	MM, leukemia, glioblastoma, mesothelioma, HCC, breast and bladder cancers
GCS-100	Loss of mitochondrial membrane integrity, release of cytochrome c and SMAC/DIABLO into the cytosol	Induction of caspases 9, 3, and 8, ↓ NF-kB, ↓VEGF, ↓ Bcl-2	MM, CLL, DLBCL, colorectal, pancreatic, prostate, renal cancers
LBH589	↑ Mitochondrial membrane permeability, release of cytochrome c, AIF and EndoG into the cytosol	Induction of caspases 9 and 3, ↑ APAF-1	MM, CTCL, DLBCL, AML, HL, breast, colon, prostate, pancreatic, ovarian, esophageal squamous cell cancers
EPED3	Loss of mitochondrial membrane integrity, release of cytochrome c into the cytosol	Inhibition of topoisomerase II, induction of caspases 8, 9, and 3	MM, APL, T cell leukemia, melanoma, CNS, thyroid, breast, lung, ovarian cancers
AT-101	Mitochondrial membrane depolarization, release of cytochrome c into the cytosol	↑ Bax/Bcl2 ratio, induction of caspases 3 and 9	MM, MCL, lung cancer
KD5170	Loss of mitochondrial membrane integrity, release of cytochrome c, SMAC/DIABLO and AIF into the cytosol	Induction of caspases 3, 8, and 9, inhibition of Bcl2/Bcl-XL	MM, CTCL, MCL, CLL, colorectal, NSCLC, prostate cancer
BV6	Loss of mitochondrial membrane integrity, release of cytochrome c and SMAC/DIABLO into the cytosol, induced degradation of IAPs	TRAIL-induced cell death, induction of caspases	MM, AML, CLL, glioblastoma, HCC
LBW242	Loss of mitochondrial membrane integrity, release of cytochrome c and SMAC/DIABLO into the cytosol	Induction of caspases 8, 9, and 3	MM, neuroblastoma, glioma, breast, renal cancers

↑: increased concentrations ↓: reduced concentrations.

## Abbreviations

2ME	2-methoxyestradiol
ROS	Reactive Oxygen Species
c-JNK	c-Jun N-terminal Kinase
MKK4/7	Mitogen-activated protein Kinase Kinase 4/7
MM	Multiple Myeloma
AIF	Apoptosis Inducing Factor
EndoG	Endonuclease G
AML	Acute Myeloid Leukemia
CNS	Central Nervous System
OXPHOS	Oxidative Phosphorilation
Nrf-2	Nuclear factor erythroid 2-Related Factor 2
VEGF	Vascular Endothelial Growh Factor
Bcl-2	B-cell Lymphoma 2
Evo	Evodiamine
Cdc2	Cyclin-dependent protein kinase
IAPs	Inhibitors of Apoptosis
NF-kB	Nuclear Factor kappa light chain enhancer of activated B cells
HSP70B’	Heat Shock Protein 70B’
HSP40	Heat Shock Protein 40
HO-1	Heme Oxygenase 1
WM	Waldenstrom’s Macroglobulinemia
DLBLC	Diffuse Large B Cell Lymphoma
UPS14	Ubiquitin carboxyl-terminal hydrolase 14
MCL	Mantle cell Lymphoma
ATP	Adenosine triphosphate
NAD	Nicotinamide adenine dinucleotide
CML	Chronic Myeloid Leukemia
RNS	Reactive Nitrogen Specie
TLR4	Toll-like receptor 4
SMAC/DIABLO	Second Mitochondria-derived Activator of Caspases/Direct Inhibitor of Apoptosis-Binding protein with low isoelectric point
BAx	Bcl-2 Associated X
HCC	Hepato cellular carcinoma
CLL	Chronic Lymphocytic Leukemia
APAF-1	Apoptotic Protease Activating Factor-1
CTCL	Cutaneous T Cell Lymphoma
HL	Hodgkin Lymphoma
APL	Acute Promyelocytic Leukemia
NSCLC	Non-Small Cell Lung Cancer
TRAIL	TNF-Related Apoptosis-Inducing Ligand
